# Workforce estimate to treat mental disorders in the Kingdom of Saudi Arabia

**DOI:** 10.1186/s12960-024-00929-6

**Published:** 2024-07-16

**Authors:** Eileen Lee, Tim A. Bruckner, Mohammad Alluhidan, Adwa Alamri, Abdulhameed Alhabeeb, Ziad Nakshabandi, Mohammed M. J. Alqahtani, Christopher H. Herbst, Mariam M. Hamza, Nahar Alazemi

**Affiliations:** 1 The World Bank, 701 18th St NW, Washington, DC 20006 USA; 2 AMBOSS GmbH, Torstrasse 19, 10119 Berlin, Germany; 3 University of California, Irvine, Health, Society and Behavior, Irvine, CA 92697-3957 USA; 4 Saudi Heath Council, Olaya St, As Sahafah, 13315 Riyadh, Saudi Arabia; 5 National Center for Mental Health, Al Mathar Ash Shamali, 12332 Riyadh, Saudi Arabia; 6 Saudi Commission for Health Specialties Central Province, Laysen Valley, Umm Alhammam Algharbi District, 7892 King Khalid Branch Road, 12329 Riyadh, Saudi Arabia; 7 King Khalid University, 6HX6+33Q, 62529 Abha, Saudi Arabia

**Keywords:** Mental health, Workforce planning, Saudi Arabia, Health workforce delivery, Health workforce shortage

## Abstract

**Background:**

Mental, neurological, and substance abuse (MNS) disorders describe a range of conditions that affect the brain and cause distress or functional impairment. In the Middle East and North Africa (MENA), MNS disorders make up 10.88 percent of the burden of disease as measured in disability-adjusted life years. The Kingdom of Saudi Arabia (KSA) is one of the main providers of mental health services and one of the largest contributors to mental health research in the region. Within the past decade, mental health resources and services has increased.

**Methods:**

We employ a needs-based workforce estimate as a planning exercise to arrive at the total number of psychiatrists, nurses, and psychosocial care providers needed to meet the epidemiological need of mental health conditions of the population of KSA. Estimates for a potential mental health workforce gap were calculated using five steps: Step 1—Quantify target population for priority mental health conditions. Step 2—Identify number of expected cases per year. Step 3—Set target service coverage for each condition. Step 4—Estimate cost-effective health care service resource utilization for each condition. Step 5—Estimate service resources needed for each condition.

**Results:**

The planning exercise indicates an epidemiologic need for a total of 17,100 full-time-equivalent (FTE) health care providers to treat priority MNS disorders. KSA appears to have a need-based shortage of 10,400 health workers to treat mental disorders. A total of 100 psychiatrists, 5700 nurses, and 4500 psychosocial care providers would be additionally needed (that is, above and beyond current levels) to address the priority mental health conditions. The shortfall is particularly severe for nurses and psychosocial workers who make up 98.9 percent of the shortfall. This shortage is substantial when compared to other high-income countries. Overall, the workforce needed to treat MNS conditions translates to 49.2 health workers per 100,000 population.

**Conclusion:**

Challenges to addressing the shortfall are Saudi specific which includes awareness of cultural customs and norms in the medical setting. These challenges are compounded by the lack of Saudi nationals in the mental health workforce. Saudi nationals make up 29.5 percent of the physician workforce and 38.8 percent of the nursing workforce. Policymakers and planners supplement this shortfall with non-Saudi providers, who must be mindful of Saudi-specific cultural considerations. Potential solutions to reducing the shortfall of mental health care workers includes nurse task shifting and training of general practitioners to screen for, and treat, a subset of MNS disorders.

## Introduction

Mental, neurological, and substance abuse (MNS) disorders describe a range of conditions that affect the brain and cause distress or functional impairment. MNS disorders include mood disorders (e.g., bipolar disorder and depression), behavioral disorders (e.g., conduct disorder), and developmental delays (e.g., autism spectrum disorder). Globally, almost one in five people would meet the diagnostic criteria for MNS disorders within the past 12 months [1]. When considered across a lifetime, this rate increases to one in every three people [1].

In the Middle East and North Africa (MENA), MNS disorders make up 10.88 percent of the burden of disease as measured in disability-adjusted life years (DALYs) [2].^1^ Persons with MNS disorders experience, on average, a 15–30 percent lower life expectancy [3] due to comorbidity with substance abuse as well as mental and physical conditions. Individuals with MNS disorders also face external challenges, some of which begin before the detection of disease and others after treatment. Patients with MNS disorders are more likely to experience barriers to accessing medical care (including difficulty with accessing physical health services) [4]. This can impede screening, diagnosis, and treatment of other underlying physical conditions [5].

Furthermore, the connection between MNS disorders and premature death from cardiovascular disease and cancers is well documented [6–8]. Patients with bipolar disorder and depression, on average, experience lower rates of preventive screening which can result in a recurring cycle of disease and disability. For example, bipolar patients who are obese have more bipolar episodes, episodes of longer duration, shorter times between episodes, and more suicide attempts than do non-obese patients [9]. These examples illustrate the individual, environmental, and systemic risk factors that pose complex challenges for health care providers. When unaddressed, these risk factors can amplify the potential sequelae of MNS disorders on population health [10]. Addressing these issues requires removing barriers to seeking help.

In the MENA region, the Kingdom of Saudi Arabia (KSA) is one of the main providers of mental health services and one of the largest contributors to mental health research [11, 12]. Within the past decade, the accessibility and availability of mental health resources (including staff, hospital beds, and associated resources used for inpatient and outpatient visits) has increased substantially [13–15]. Research has also increasingly quantified and classified mental illness in primary care settings [16, 17]. More recently, a national study was published which assessed the population prevalence and correlates of mental health conditions in KSA [18].

KSA has embarked on the Saudi Health Transformation Program as part of the broader Vision 2030 reforms, KSA’s ambitious blueprint for economic and social transformation. Vision 2030 aims to diversify its economy, reduce dependence on oil, and improve the quality of life for its citizens. KSA’s healthcare transformation program aims to enhance the quality, accessibility, and efficiency of its healthcare system. The reform of the primary care system includes an integrated, family-centered approach with new clinical pathways, screening for chronic diseases, and integration of mental health services. The transformation is characterized by significant investments in technology and capacity-building, leading to improved access to services and a more comprehensive care delivery model [19]. In addition, the focus on mental health is crucial, given the rising prevalence of mental disorders globally and in Saudi Arabia. Only 1.2% of physicians in Saud Arabia are psychiatrists [20]. Additionally, as of 2022, there were 1052 psychotherapists across the entire country serving more than 34 million [20].

Despite KSA’s developments in recent decades, significant social challenges remain. Saudi-specific social and cultural factors need to be considered as part of service delivery for mental health conditions [21, 22]. One of these factors involves the perception of mental illness and the role of the family in Saudi culture. There is a belief that mental disorder is the result of supernatural causes, weak faith, or weakness of character [22]. Lack of public awareness of the origin, treatment of, and functional capability of patients with mental disorders [23, 24] means that affected individuals may attempt to hide their disorder or be unwilling to seek help if having a condition would reflect poorly on their family [25, 26]. This could delay much-needed treatment and result in further progression of disease.

The problem of stigma poses multiple challenges for health care practitioners, including demand-side patient underutilization of mental health services [27], limited screening and detection (patient somatization of mental health conditions) [27, 28] and a shortage of the supply of Saudi mental health professionals [29]. Underutilization of mental health services in the medical system means that persons with MNS disorders instead turn to faith healers and traditional remedies [24]. Furthermore, special training for mental health screening has been recommended for primary care providers, who serve as the first point of access to the treatment of MNS disorders within the health care system [30]. Lastly, the negative perception of mental illness and psychiatry means that there is a shortage of mental health professionals in KSA [29].

In addition, the COVID-19 pandemic has led to an unanticipated increase in the demand for mental health services. During the lockdown, up to 23.6% of survey respondents reported a moderate or severe psychological impact with severe symptoms of stress [31]. People with and without psychiatric illnesses were more likely to show higher levels of PTSD, anxiety, depression and stress [31]. These symptoms were more severe for those with existing mental conditions. This situation has exacerbated existing mental health service delivery gaps (especially among hard to reach and rural populations).

The Saudi Health Council projected the 2030 needs-based demand and supply for physicians and nurses. The projected need for full-time equivalent (FTE) physicians and nurses in Saudi Arabia by 2030 ranges from 60,000 to 112,000, depending on various assumptions. This range translates to densities of 1.64 to 3.58 per 1,000 population. When considering both Saudi and non-Saudi nationals, Saudi Arabia seems to have a sufficient number of health workers to meet epidemiological needs. However, there may be a shortage if only Saudi nationals are counted, especially when excluding diploma nurses without a college degree in nursing. We anticipate that a key outcome of this planning exercise is for KSA to assess whether the current workforce levels and training capacity of Saudi nationals could sufficiently meet the mental health needs of the population.

In this paper, we estimate the gap between the current supply of mental health workers and the number needed to adequately treat the population for KSA by estimating the current prevalence of mental health disorders and needed resources for treatment. The analysis employs an epidemiologic need-based model of MNS disorders in KSA to estimate the need for mental health workers. This need-based model departs from most economic demand-based estimates in several ways. First, we use the population-based prevalence of MNS disorders (based on epidemiologic studies) as the cornerstone of its estimate. This approach differs from traditional economic models which conflate demand with need and assume that population need is reflected by current help-seeking behaviors and the price of health care [32, 33]. Second, the need-based model does not consider either governmental or patient willingness-to-pay. This approach, therefore, serves as a planning exercise for policymakers in estimating, in an ideal scenario, the number of health workers that would be needed to treat the prevalent conditions in the population. Our approach is based on guidance from the World Health Organization [34], aligns closely with prior work [35], and has been used in both MENA and other regions [35–37]. For these reasons, our approach permits direct comparisons of KSA’s results with those of other countries.

## Methodology

Estimates for a potential mental health workforce gap in KSA were calculated using five steps [38]: *Step 1*—Quantify target population for priority mental health conditions: (a) Obtain age-specific population prevalence data. (b) Identify age-specific population counts. *Step 2*—Identify number of expected cases per year. a) Multiply the age-specific prevalence of priority health conditions by population size to arrive at the total number of (age-specific) cases. *Step 3*—Set target service coverage for each condition. *Step 4*—Estimate cost-effective health care service resource utilization for each condition. *Step 5*—Estimate service resources needed for each condition. (a) Calculate full-time-equivalent (FTE) staff needed for each treatment setting at target coverage. (b) Assign staffing ratios based on treatment setting needs.

### Step 1—Quantify target population for priority mental health conditions.

As defined in the World Health Organization (WHO)’s Mental Health Gap Action Programme (mhGAP) [34], we focused on 11 priority mental health conditions: depression, bipolar disorder, schizophrenia, dementia, alcohol use, drug use, suicide, epilepsy, and intellectual disabilities, and developmental and behavioral disorders in children. Prioritization for these conditions was determined by assessing their impact on cost-effectiveness, affordability, and feasibility of treatment [34, 35]. We focused on these conditions because of their large disability burden and the availability of cost-effective treatment service interventions that can be administered by a health worker.

After identifying the priority conditions, the target population with these conditions was quantified in two parts. The first part consisted of identifying the age-specific population prevalence of MNS disorders using five data sources: the Saudi National Mental Health Survey [39], the WHO Global Observatory Database [40], the WHO World Alzheimer’s Report [42 primary research, and the IMHE Global Burden of Disease (GBD) [38].

#### Obtain age-specific population prevalence data

We prioritized studies conducted in KSA or MENA that sampled from a near-complete population sampling frame that includes households and individuals from population registers and/or censuses and lists of children from schools. Of the studies considered, case–control designs without 100 percent geographic representativeness selection were preferred over non–case–control studies from a more geographically representative population.

Priority was given to studies with stronger sampling methods and research design, and that included a medical professional to validate a diagnosis of an MNS disorder. We used results from studies that employed a nationally and geographically representative sampling of households and participant recruitment in addition to a case–control design^2^ [39]. As a second option, we used prevalence estimates from non-nationally representative case–control studies. There were three studies that met these criteria including regional meta-analyses [39, 40] and the primary source literature [41]. This rank preference of data sources aligns with WHO guidance [42].

In the case of assessing prevalence of developmental, behavioral, and emotional conditions in children, we reviewed two studies that utilized case–control design where survey instruments included assessments of multiple perspectives including parents, teachers, and children [43, 44]. However, when compared to GBD estimates [38], the estimates from primary sources [43, 44] differed greatly and ranged from being 4.7 times lower for childhood intellectual disabilities to 13.9 times higher for childhood emotional disorders.^3^ These differences were deemed to arise from study design challenges rather than reflecting a true difference in the prevalence of these disorders across regions. Therefore, for these conditions, we deferred to GBD estimates. In the case of dementia, we utilized a meta-analysis study [45]. Table 1 summarizes the studies that were included in this study and their relative ranking in priority of the aforementioned criteria. (See appendix A for a full description of data sources.) Table 2 summarizes the results of the literature review prevalence estimates for each priority mental health condition. We also note that, in light of the lack of convergence of prevalence estimates across studies, we provide a few sensitivity tests (see Appendix D) of mental health worker need under various prevalence scenarios. In addition, whereas we use point estimates in presenting results, we acknowledge that several prevalence estimates used to arrive at workforce results are measured imprecisely.

**Table 1:** Overview of selection criteria for estimates of prevalence for priority health conditions in 2020

Rank	Research type	Data Source	Sampling methodology	Mental health conditions
1	Population survey	World Mental Health (WMH) Survey [39]	Multistage household probability sample with case–control design Fully structured diagnostic interview using the World Mental Health Composite International Diagnostic Interview (WMH—CIDI) Part 1—Core diagnostic assessment administered by trained interviewer Part 2—Respondents who meet criteria for any disorder in Part 1 + subsample of ~ 25% of non-criteria respondents	Depression, alcohol use disorder, and drug use disorders
2	Meta analysis	World Alzheimer’s Report [45]	Systematic literature review conducted via PubMed/Medline for population-based studies among people 60 + years^a^	Dementia
3	Primary research	Al Rajeh et al. [41]	Community sample of *N* = 23,700 Saudis in Thugbah with case control Part 1—Structured interview was carried out by trained interviewer using WHO protocol for detecting neurological disorders Part 2—Individuals identified as having a neurological disorder were evaluated by a neurologist	Epilepsy
4	Simulation	WHO Global Health Observatory [40]	Statistical modeling performed using data from regional health observatories and international agencies with consultation from member states and experts. Includes household surveys, civil registration of vital events, and institution-based sources (administrative and health facilities)	Suicidal ideation
5	Simulation	IHME Global Burden of Disease [45]	Statistical modeling incorporates data from censuses, national surveys, primary research, births, and vital registration	Bipolar disorder,b child intellectual and development disorders, child conduct/behavioral disorders, and child emotional disorders

Source: Original table for publication

GBD: Global Burden of Disease; WMH-CDI: World Mental Health Composite International Diagnostic Interview

^a^Studies for the Middle East and North Africa relied on data from expert consensus panels (2005) and studies from Egypt and Turkey [43, 44, 46–50].

^b^For bipolar disorder, we initially utilized estimates from WMH Surveys. However, these estimates resulted in projections of workforce needs that were 1.5 times higher than historical estimates for low- and middle-income countries [35]. These substantially higher estimates would significantly impact the ability to compare current and historical estimates in low- and middle-income countries. Therefore, we utilized GBD estimates for bipolar disorder

**Table 2:** Prevalence (%) of Priority Mental Health Conditions in 2020

Age	0–14	15–34	35–49	50–64	65 +	Total (%)
Schizophreniaa	n.a	0.33%	0.53%	0.43%	0.24%	0.41
Bipolar disorder^a^	n.a	1.11%	1.09%	1.04%	0.69%	1.07
Depression^b^	n.a	3.39%	4.13%	4.17%	3.51%	3.80
Suicidal ideation^c^	n.a	0.07%	0.09%	0.10%	0.15%	0.07
Dementia^d^	n.a	n.a	n.a	0.10%	2.44%	0.10
Alcohol use disorder^b^	n.a	0.25%	0.29%	0.16%	0.10%	0.20
Other drug use disorder^b^	n.a	3.29%	2.36%	1.32%	0.63%	1.90
Epilepsy^e^	0.76%	0.54%	0.32%	0.23%	0.56%	0.50
Childhood intellectual disabilities^a^	1.36%	n.a	n.a	n.a	n.a	1.36
Childhood conduct/behavioral disorders^a^	2.75%	n.a	n.a	n.a	n.a	2.75
Childhood emotional disorders^a^	0.69%	n.a	n.a	n.a	n.a	0.69

This table is not adjusted for comorbidity of conditions. Definition of mental health conditions. Schizophrenia = cases that meet ICD-10 criteria for schizophrenia only. Bipolar disorder = cases that meet ICD-10 criteria for bipolar disorder only. Depression = cases that meet DSM-IV criteria for major depressive disorder only with clinical follow-up. Suicidal ideation = WHO Global Health Observatory suicide death rate multiplied by factor of 20 [51, 52]. Dementia = cases that meet ICD-10 criteria for dementia multiplied by a 0.5 correction factor [53]. Alcohol use disorder = cases that meet DSM-IV criteria for alcohol dependence and alcohol use disorder with clinical follow-up. Other drug use disorders = cases that meet DSM-IV criteria for substance (non-alcohol) dependence and substance (non-alcohol) use disorders with clinical follow-up. Epilepsy = cases that meet the International League Against Epilepsy definition for seizures (within past 6 months) with clinical and electroencephalographic follow-up. Childhood intellectual disabilities = cases that meet the ICD-10 criteria for pervasive developmental disorder including autism. Childhood conduct and behavioral disorders = cases that meet ICD-10 criteria for attention deficit hyperactivity disorder, conduct disorder, and oppositional defiant disorder. Childhood emotional disorders = cases that meet ICD-10 criteria for depressive disorders (major depression and dysthymia) and mania (bipolar disorder)

DSM-IV: Diagnostic and Statistical Manual of Mental Disorders, fourth edition**;** ICD-10: International Classification of Diseases, Tenth Revision; n.a.: not applicable

Sources: ^a^[54]

^b^[39]

^c^[40]

^d^[45]

^e^[41]

#### Identify age-specific population counts

The relevant age-specific population counts were identified using UN Population Estimates (Table 3).

**Table 3:** Population by age group, 2020

Age group	Count
0–14	8,597,715
15–34	11,011,479
35–49	9,951,215
50–64	4,035,509
65 +	1,217,949
Total	34,813,867

Source: [55]

### Step 2—Identify the number of expected cases per year

This age-specific prevalence of priority health conditions was applied to UN population estimates for KSA (Table 3) to arrive at the total number of cases within the population.

#### Multiply the age-specific population prevalence of priority health conditions by population size

For example, the estimated prevalence of bipolar disorder is 1.11 percent for persons ages 15–34,^4^ which yields 122,510 cases.

$$prevalence \times population= number\, of\, expected\, cases$$

$$1.11\% \times \text{11,011,479}= \text{122,510}$$

### Step 3—Set target coverage for the target populations for each condition

Targets for health service coverage quantify the service resource allocation and delivery that is feasible for the population affected by a given MNS disorder. Target rates for coverage of each disorder were obtained via literature review [56–61]. Target coverage rates were set higher (for example, 80 percent) for conditions that have a higher disability, visibility, and vulnerability—such as schizophrenia and bipolar disorder. Targets were lower (for example, 25 percent) for conditions that are challenging to detect and/or are less likely to involve the patient seeking care. For example, alcohol use disorder [57] remains relatively “hidden” in the sense that persons with this disorder are not likely to be identified by a clinician and not likely to seek care. We refer the reader to Table 10 in appendix for details. In addition, we note the important caveat that, in resource-constrained contexts, attainment of these target treatment coverage rates may not be feasible or achievable. On-the-ground realities may, for instance, preclude implementation of these treatment coverage rates. We also note that altering of target coverage rates substantially changes estimates of workforce need; for a much more detailed discussion and sensitivity analysis of treatment coverage rates, see Bruckner et al. [35].

Defining the target population effectively determines who, based on epidemiologic need, would require access to health services. This need-based target differs from other benchmarks [62] that determine need based on desired equity goals (for example, universal health coverage in which all individuals receive the help that they need, when they need it, without financial hardship, and without barriers to accessibility). The need-based target assumes no cost barriers to care and diverges from other benchmarks in that it does *not* assume universal treatment coverage of 100 percent for all persons with MNS disorders.

$$number\, of\, expected\, cases \times target\, coverage=target population$$

The target coverage for bipolar disorder is 80 percent, which means that (for example) the target population for patients with bipolar disorder ages 15–34 is 98,008 persons.

$$\text{122,510} \times 80\%=\text{98,008} persons$$

### Step 4—Estimate cost-effective health care service resource utilization for each condition

The health care service delivery model in the mhGAP estimates the FTE staff needed to effectively deliver mental health interventions for low- and middle-income countries. The required inputs—health care worker, rate of use, and facility type—for staffing calculations vary for each of the priority health conditions in accordance with the literature [51]. See Table 11 in appendix B for more details.

The total annual outpatient visits and inpatient bed-days that would be expected for the target population at the specified target service coverage rates (see Tables 11 and 12 in appendix B) are used to estimate FTE. Assuming that health care workers provide 11 consultations per day with 225 working days per year, 176,414 outpatients visits for patients with bipolar disorder (ages 15–34 only) per year will require 71 hospital outpatient FTE employees. Within the outpatient primary care setting, we assume that psychosocial care providers will perform 77.50 percent of the tasks and that nursing care providers will perform 20.83 percent. This leaves psychiatrists and specialists with the remaining 1.67 percent of the tasks. Following this distribution, 1 psychiatrist, 15 nurses, and 55 psychosocial care providers are needed to treat the target population for patients with bipolar disorder ages 15–34.^5^

### Step 5—Estimate service resources needed for each condition

Next, estimates of service resources were calculated for each of the priority conditions. This was assessed in outpatient visits (for treatment settings in day care and primary care) and inpatient bed-days (for treatment settings in acute care and long stay/residential care).

$$\text{FTE needed}=\frac{\text{service utilization }(\text{e}.\text{g}.,\text{ visits per year})}{(\text{consultations per day }\times \text{ working days per year})}$$

Using the total number of outpatient visits and inpatient bed-days, we applied the calculations for staffing patterns to each health care setting. The final step consists of assigning staffing ratios based on treatment settings.

Whereas our workforce planning tool provides point estimates of the mental health workforce needed to treat the priority MNS disorders, we caution the reader against assuming high precision of these estimates. Our workforce figures would vary broadly if we instead changed assumptions or assumed different structures of underlying input data (e.g., different prevalence). For this reason, we include a short suite of sensitivity analyses that use different assumptions (see Appendix D) and remind the reader that there is a range of possible values around our point estimates.

## Results

### Prevalence and cases

**T**he number of cases in need of treatment were calculated based on the prevalence of MNS disorders. Table 4 shows the total target cases that will require treatment by age group. The total number is 1,153,100 (rounded^6^) or 3312 per 100,000, for the priority mental health conditions. Of the target population of persons with MNS disorders, 48 percent of the target population is estimated to suffer from depression or bipolar disorder (see Table 5). The third largest group is other drug use disorders at 22.47 percent.

**Table 4:** Target population that requires treatment for priority mental health conditions in 2020

Age	0–14	15–34	35–49	50–64	65 +	Total
Schizophrenia^a^	n.a	28,800	42,000	13,800	2400	86,900
Bipolar disorder^a^	n.a	98,000	86,800	33,400	6700	225,000
Depression^b^	n.a	123,200	135,600	55,500	14,100	328,400
Suicidal ideation^c^	n.a	1900	2100	1000	400	5500
Dementia^d^	n.a	0	0	2000	14,900	16,900
Alcohol use disorder^b^	n.a	4900	4700	700	100	10,400
Other drug use disorder^b^	n.a	154,200	88,400	15,300	1200	259,100
Epilepsy^e^	52,400	47,500	25,600	7500	5500	138,500
Childhood intellectual disabilities^a^	23,300	n.a	n.a	n.a	n.a	23,300
Childhood conduct/behavioral disorders^a^	47,200	n.a	n.a	n.a	n.a	47,200
Childhood emotional disorders^a^	11,800	n.a	n.a	n.a	n.a	11,800
Total target cases	134,700	458,600	385,100	129,300	45,300	1,153,100
Total cases per 100,000 population	1567	4165	3870	3204	3718	3312

This table shows the target population for each condition unadjusted for comorbidity. Definition of mental health conditions. *Schizophrenia* = cases that meet ICD-10 criteria for schizophrenia only. *Bipolar disorder* = cases that meet ICD-10 criteria for bipolar disorder only. *Depression* = cases that meet DSM-IV criteria for major depressive disorder only with clinical follow-up. *Suicidal ideation* = WHO Global Health Observatory suicide death rate multiplied by factor of 20 [51, 52]. *Dementia* = cases that meet ICD-10 criteria for dementia multiplied by a 0.5 correction factor [53]. *Alcohol use disorder* = cases that meet DSM-IV criteria for alcohol dependence and alcohol use disorder with clinical follow-up. *Other drug use disorders* = cases that meet DSM-IV criteria for substance (non-alcohol) dependence and substance (non-alcohol) use disorders with clinical follow-up. *Epilepsy* = cases that meet the International League Against Epilepsy definition for seizures (within past 6 months) with clinical and electroencephalographic follow-up. *Childhood intellectual disabilities* = cases that meet the ICD-10 criteria for pervasive developmental disorder including autism. *Childhood conduct and behavioral disorders* = cases that meet ICD-10 criteria for attention deficit hyperactivity disorder, conduct disorder, and oppositional defiant disorder. *Childhood emotional disorders* = cases that meet ICD-10 criteria for depressive disorders (major depression and dysthymia) and mania (bipolar disorder)

DSM-IV: Diagnostic and Statistical Manual of Mental Disorders, fourth edition; ICD-10: International Classification of Diseases, Tenth Revision; n.a.: not applicable

Sources: ^a^[54]

^b^[39]

^c^[40]

^d^[45]

^e^[41]

**Table 5:** Percent of target population that requires treatment for priority mental health conditions (% within age groups) in 2020

Age	0–14	15–34	35–49	50–64	65 +	Total (%)
Schizophrenia^a^	n.a	6.30%	10.90%	10.70%	5.20%	7.50
Bipolar disorder^a^	n.a	21.40%	22.50%	25.90%	14.90%	19.50
Depression^b^	n.a	26.90%	35.20%	42.90%	31.20%	28.50
Suicidal ideation^c^	n.a	0.40%	0.50%	0.80%	0.90%	0.50
Dementia^d^	n.a	0.00%	0.00%	1.60%	32.80%	1.50
Alcohol use disorder^b^	n.a	1.10%	1.20%	0.60%	0.30%	0.90
Other drug use disorder^b^	n.a	33.60%	23.00%	11.80%	2.60%	22.50
Epilepsy^e^	38.90%	10.40%	6.60%	5.80%	12.00%	12.00
Childhood intellectual disabilities^a^	17.30%	n.a	n.a	n.a	n.a	2.00
Childhood conduct/behavioral disorders^a^	35.00%	n.a	n.a	n.a	n.a	4.10
Childhood emotional disorders^a^	8.80%	n.a	n.a	n.a	n.a	1.00

This table shows the target population for each condition unadjusted for comorbidity. Definition of mental health conditions. *Schizophrenia* = cases that meet ICD-10 criteria for schizophrenia only. *Bipolar disorder* = cases that meet ICD-10 criteria for bipolar disorder only. *Depression* = cases that meet DSM-IV criteria for major depressive disorder only with clinical follow-up. *Suicidal ideation* = WHO Global Health Observatory suicide death rate multiplied by factor of 20 [51, 52]. *Dementia* = cases that meet ICD-10 criteria for dementia multiplied by a 0.5 correction factor [53]. *Alcohol use disorder* = cases that meet DSM-IV criteria for alcohol dependence and alcohol use disorder with clinical follow-up. *Other drug use disorders* = cases that meet DSM-IV criteria for substance (non-alcohol) dependence and substance (non-alcohol) use disorders with clinical follow-up. *Epilepsy* = cases that meet the International League Against Epilepsy definition for seizures (within past 6 months) with clinical and electroencephalographic follow-up. *Childhood intellectual disabilities* = cases that meet the ICD-10 criteria for pervasive developmental disorder including autism. *Childhood conduct and behavioral disorders* = cases that meet ICD-10 criteria for attention deficit hyperactivity disorder, conduct disorder, and oppositional defiant disorder. *Childhood emotional disorders* = cases that meet ICD-10 criteria for depressive disorders (major depression and dysthymia) and mania (bipolar disorder)

DSM-IV: Diagnostic and Statistical Manual of Mental Disorders, fourth edition; ICD-10: International Classification of Diseases, Tenth Revision; n.a.: not applicable

Sources: ^a^[54]

^b^[39]

^c^[40]

^d^[45]

^e^[41]

### Needed consultations

It is estimated that approximately 7,084,800 regular visits per year are needed for the selected MNS conditions with bipolar disorder, depression, and drug use disorders contribute to the highest proportion of total outpatient visits. Table 6 shows the expected annual outpatient and inpatient resources needed (as measured in visits and days) to manage the target cases of priority mental health conditions. Regular (outpatient) visits account for 71.16 percent of the total outpatient visits. This equates to 7,084,800 regular visits per year (or 20,351 per 100,000 population). Day care visits make up 28.84 percent of total outpatient visits (or 8200 visits per 100,000 population). Children aged 0–14 with MNS conditions are estimated to spend 97.2 percent of their visits and days in outpatient care. This distribution changes for patients aged 15 and over who are estimated to spend around 60 percent of their visits in outpatient care.

**Table 6:** Total expected annual outpatient visits and inpatient days for target cases for priority health conditions in 2020

Age group	Outpatient				Inpatient			
	Day care visits		Regular visits		Acute days		Community residential days	
	Visits	Per 100,000 population	Visits	Per 100,000 population	Days	Per 100,000 population	Days	Per 100,000 population
0–14	11,600	135	830,000	9654	0	0	24,300	282
15–34	1,199,000	10,888	2,734,700	24,835	228,300	2073	2,287,600	20,775
35–49	1,140,500	11,461	2,410,500	24,223	231,700	2329	1,847,200	18,563
50–64	405,900	10,058	843,200	20,894	85,000	2106	616,800	15,285
65 +	114,200	9378	266,500	21,881	16,400	1347	501,000	41,136
Total	2,871,100	8247	7,084,800	20,351	561,500	1613	5,277,000	15,158

Source: Calculations for visits and day-beds performed using data from refs. [39–41, 45, 54, 57]. This table is adjusted for comorbidity of conditions and treatable diseases. See details in appendix C. The calculations for the visits are rounded to the nearest 100

^a^Regular visits includes hospital outpatient visits, primary health care (PHC) treatment, and PHC screenings

In Table 7, expected annual outpatient visits and inpatient days are shown by condition. Bipolar disorder, depression, and drug use disorders contribute to the highest proportion of total outpatient visits. The treatment models for depression, drug use disorders, and bipolar disorder assume that regular visits (90.5 percent, 75.9 percent, and 56.1 percent, respectively) will make up the majority of outpatient care. Bipolar disorder has a prevalence of 1.07 percent and comprises over one-third (38.6 percent) of total outpatient visits. This is due to the high treatment service coverage of 80 percent and the high average service utilization for both outpatient visits (see appendix B). Compared to bipolar disorder, depression has a higher population prevalence at 3.80 percent and contributes to about half of the number of outpatient visits, which is 17.4 percent of the total outpatient visits. This is due to its relatively lower treatment coverage (33 percent) and the average service utilization for depression.

**Table 7:** Total expected annual outpatient visits and inpatient days for target cases by priority mental health condition in 2020

Condition	Total outpatient visits			Total inpatient days		
	Day care	+ Regular visits	= Total outpatient visits per condition	Acute	+ Residential care	= Total inpatient days per condition
Bipolar disorder	1,687,400	2,159,900	3,847,300	405,000	1,957,400	2,362,400
Depression	164,200	1,566,700	1,730,900	0	239,800	239,800
Other drug use disorders	310,900	979,400	1,290,300	0	1,813,700	1,813,700
Schizophrenia	652,000	834,600	1,486,600	157,000	756,300	912,800
Epilepsy	0	969,200	969,200	0	34,600	34,600
Conduct/behavioral disorders in children	5700	330,000	335,700	0	2600	2600
Intellectual disability in children	0	77,000	77,000			0
Dementia	42,300	76,100	118,400	0	456,500	456,500
Emotional disorders in children	5900	56,300	62,200	0	8600	8600
Suicide ideation	2700	25,600	28,300	0	6400	6400
Alcohol use disorder	0	10,100	10,100	0	1000	1000
Total	2,871,100	7,084,800	9,956,000	561,500	5,277,000	5,838,400

Source: Calculations for visits and day-beds performed using data from refs. [39–41, 45, 54, 57]. The calculations for the visits are rounded to the nearest 100

Need varies significantly by age group due to the significant variation by age group in inpatient and outpatient visits needed. Children ages 0–14 have the lowest rates of total outpatient visits and inpatient bed usage. This low rate can be attributed to the lower target treatment coverage (20 percent) for disorders in children and the lower service utilization rate for childhood conduct/behavioral disorder and intellectual and developmental disorders. Patients ages 35–49 have the highest rate of outpatient regular visit usage, at 24,900 visits per 100,000 population. This group has the highest prevalence of schizophrenia, alcohol use disorder, and drug use disorders, as well as the second highest rate of depression. Patients who are ages 65 + have the highest rate of inpatient bed-day usage, at 41,100 bed-days per 100,000 population for community residential care. The primary conditions affecting this group are depression and dementia, at 3.51 percent and 2.44 percent, respectively, of which the treatment model for dementia consists primarily of residential care.

Bipolar disorder and other drug use disorders have the highest number of total inpatient days. For bipolar disorder, 38.0 percent of the total visits and days will be spent in inpatient care of which 82.9 percent will be spent in residential care and 11.2 percent will be spent in acute day care. For other drug use disorders, 58.4 percent of the total patient visits and days will be spent in inpatient residential care. Residential care makes up the majority of total inpatient days with bipolar disorder contributing to the largest proportion of annual residential care bed-days. Table 7 shows that residential care makes up the overwhelming majority (90.38 percent) of total inpatient days (5,277,000 bed-days) and acute treatment makes up 9.84 percent (or 561,500 bed-days). Bipolar disorder contributes to the largest proportion of annual residential care bed-days at 37.09 percent, or 1,957,400 bed-days.^7^ Other drug use disorders have a prevalence of 1.90 percent and target coverage of 50 percent. This condition makes up the second highest residential bed-days at 31.06 percent, or 1,813,700 bed-days.

Although dementia has a population prevalence of 0.10 percent, this condition is expected to contribute to 1.2 percent of total outpatient visits and 7.8 percent of total inpatient days per year. Dementia has the highest proportion of care in the inpatient setting at 79.4 percent. This high inpatient service utilization represents the high cost and debilitating toll of dementia. These figures are already adjusted for comorbidity of conditions. Within the conditions assessed, there are staff FTE and treatment optimizations that could be made if the comorbidities are identified and treatable as part of another condition’s treatment model. (See appendix C for full details.)

### Estimated staff needs

The selected conditions would require approximately a total of 9,955,900 outpatient visits and 5,838,400 inpatient visits (see Table 7). The estimated number of staff needed to treat the priority mental health conditions is 1000 psychiatrists, 9400 nurses, and 6600 psychosocial care providers would be needed to address the priority mental health conditions (see Table 8 for age-specific breakdown). This amounts to 17,100 FTE staff. KSA currently has 900 psychiatrists, 3700 psychiatric nurses, and 2100 psychosocial care providers (Table 9). A total shortfall of 10,400 mental health workers is predicted and when segmented by role, this amounts to 100 psychiatrists, 5700 nurses, and 4500 psychosocial care providers (see Table 9). The shortfall is particularly severe for nurses and psychosocial workers who make up 98.9 percent of the shortfall. This shortage is substantial when compared to other high-income countries. Overall, the workforce needed to treat MNS conditions translates to 49.2 health workers per 100,000 population.

**Table 8:** Estimated FTE staff needed to treat mental health conditions in 2020

Age group	0–14	15–34	35–49	50–64	65 +	Total
Psychiatrists						
FTE staff needed	10	447	368	124	94	1047
Per 100,000 population	0.1	4.1	3.7	3.1	7.7	3
Per 100,000 treated cases	7.4	97.6	95.7	95.7	207.8	90.5
Psychiatric nurses						
FTE staff needed	108	4003	3360	1137	794	9440
Per 100,000 population	1.3	36.4	33.8	28.2	65.2	27
Per 100,000 treated cases	80.4	872.9	872.4	879.3	1,753.50	815.4
Psychosocial care providers^a^						
FTE staff needed	279	2619	2333	797	469	6641
Per 100,000 population	3.2	23.8	23.4	19.8	38.5	18.7
Per 100,000 treated cases	207	571	606	616	1036	564
Total FTE staff needed						
FTE staff needed	397	7069	6061	2058	1357	16,943
Per 100,000 population	4.6	64.2	60.9	51	111.4	48.7
Per 100,000 treated cases	295	1541.50	1573.90	1591.40	2997.50	1469.40

Source: Calculations for FTE performed using data from refs. [39–41, 45, 54, 57]. Calculation assumes that there are 225 working days per year with 11 consultations per day [38]

FTE: full-time equivalent

^a^*Psychosocial care providers* includes social workers or psychologists

**Table 9:** Estimated FTE staff shortfall to treat mental health conditions in 2020

	Psychiatrists		Psychiatric nurses		Psychosocial care providers^a^		Total	
	*N*	Per 100,000	*N*	Per 100,000	*N*	Per 100,000	*N*	Per 100,000
FTE staff supply	900	2.7	3700	10.7	2100	6.0	6700	19.3
FTE needed	1000	3.0	9400	27.1	6600	19.1	17,100	49.2
Shortfall	100	0.3	5700	16.5	4500	13.1	10,400	29.9

Source [13]. Calculation assumes that there are 225 working days per year with 11 consultations per day [38]. The estimates for FTE and shortfall are rounded to the nearest 100

FTE: full-time equivalent

^a^*Psychosocial* *care providers* includes social workers or psychologists

## Discussion and recommendations

This paper used a need-based methodology to assess the potential shortfall of mental health workers in KSA needed to treat priority MNS conditions. The analysis, which we view as a planning exercise, employs an epidemiologic need-based model of MNS disorders in KSA to estimate the need for mental health workers. This need-based model differs from “traditional” demand-based economic models which assume that the need for care is largely reflected by help-seeking behaviors and price of care. Our model, by contrast, assumes that epidemiologic prevalence of MNS conditions serves as the starting point for assessing population need of health care treatments. Whereas WHO has recommended this strategy in the past [42], it remains underutilized as a planning tool [35–37].

A shortfall of 10,400 workers to treat mental health conditions is predicted in KSA, which means that a total of 100 psychiatrists, 5700 nurses, and 4600 psychosocial care providers would be additionally needed (i.e. above and beyond current levels) to address the priority mental health conditions. This shortfall is particularly severe for nurses and psychosocial workers (defined as social workers and psychologists) who make up 98.9 percent of the shortfall.

In addition, there is a lack of trained professionals to treat the unique needs of special populations (for example, children, adolescents, and the elderly) [14]. Due to data limitations this report does not allow for gender-specific analyses. However, it is important to note that anecdotal evidence suggests that women in KSA are more vulnerable to mental health disorders as well as face more access issues due to cultural barriers and stigma. Additionally, the scope of the report is limited to evaluation the shortfall of workers. Further studies are needed to cost the wage bill associated with such an expansion of human resources for mental health, which will likely need to be a phased expansion given its magnitude.

Whereas KSA is open to examining a wide range of task-shifting possibilities, the main task-shift that appears feasible is that among nurses. In countries that have implemented extensive task shifting, nurses can cover nearly an entire patient visit which has been demonstrably effective in expanding access to and continuity of care [63]. A systematic review of nurse task shifting for mental health specialists in primary care suggests that nurses performed the tasks typically carried out by specialists, with higher qualifications, and were able to achieve similar patient outcomes [64]. Nurse-delivered task shifting interventions were generally the most effective [64]. Short training modules that spanned two hours or up to one week were effective for shifting a variety of tasks ranging from screening, therapy, to carrying out extensive interventions [64, 65]. In addition, the Saudi health transformation program aims to enhance efficiency by introducing new cadres, such as medical coders and other allied health professionals, into the healthcare system. These additions are intended to improve healthcare quality, streamline administrative processes, and support the overall modernization of the sector.

There may be challenges to addressing the shortfall with Saudi health care workers due to stigmatized perceptions and burnout, which requires innovative training strategies. Changing the public perception of psychiatry and the perception of nursing as a profession are necessary to ensuring a sufficient supply of Saudi mental health care professionals to meet the current and future needs of the population [29]. In addition, psychiatrists in other high-income country settings report relatively more burnout than do other specialties. One alternative, which is increasingly employed in high-income countries, involves training general practitioners to screen for, and treat, a subset of MNS disorders. Telemedicine is another innovative strategy that has been used with wide ranging success to address conditions during the global COVID-19 lockdowns [66, 67]. This can be used to access difficult to reach populations, increase coverage, reduce hospitalizations, lost productivity, and increase cost effectiveness [68]. KSA may want to consider such innovative training strategies to address the shortfall of treatment options for the population with MNS disorders. In addition, innovative strategies to train staff to screen for “hidden” conditions (e.g., alcohol use) would also have the potential to successfully identify and treat MNS disorders.

This worker shortfall would likely be more severe if KSA were to focus solely on Saudi nationals. The majority (56 percent) of the health care workforce is foreign [69, 70]. Saudis make up 29.5 percent of the physician workforce [69] and 38.8 percent of the nursing workforce [70]. This composition has specific implications for mental health care providers when it comes to observing KSA-specific cultural customs and norms [71, 72]. In addition, the extent to which patient/provider concordance, in terms of Saudi national status or gender, could assist with de-stigmatizing help-seeking for MNS disorders remains unclear. Whereas the estimates provided in this report assume a specific level of help-seeking for each condition, consideration of the composition of the health care workforce, as well as public health and other efforts, could substantially affect help-seeking behavior for MNS disorders.

We remind the reader that, although we report point estimates for worker shortages, many of the data inputs to our model are measured imprecisely. For this reason, there are a range of possible values around these estimates (see Appendix D for some sensitivity analyses). We also acknowledge that the planning exercise is a simulation; the feasibility of bridging the shortfall of health workers may be low. These figures, rather, are intended to illustrate the magnitude of the shortfall and to enable policymakers to work on both short and long-term strategies to increase the mental healthcare workforce—whether it includes Saudi nationals only or a mix of internationally- and nationally- trained workers.

In a WHO 2003 book focused on planning and budgeting to deliver mental health services, the Authors provide the basis of our epidemiologic needs-based approach [42]. Unlike economic demand-based estimates which focus on revealed preferences (e.g., willingness to pay for mental health services), the needs-based approach assumes that the population-based prevalence of MNS disorders serves as the organizing principle for workforce planning. This proposed approach was novel at the time because it, at the first stage, did not necessarily constrain estimates of workforce need by that country’s current budget or their level of workforce resources. For this reason, the needs-based approach that we employ could be viewed as a visioning (or goal-setting) exercise that places the health conditions of the population as the organizing principle of workforce planning. We further acknowledge, moreover, that applications of the needs-based approach include assumptions that take into account some practical realities of treating MNS disorders. For instance, the assignment of a “target” treatment coverage rate for each disorder, which has been described in detail elsewhere [35], considers (among other aspects) stigma related to help-seeking and the likelihood of clinical detection of the condition.

The need-based estimate of worker shortfalls to treat MNS disorders in KSA relies on several assumptions, which likely result in a conservative estimate of the shortfall. Whereas these assumptions relate to each step of the model-building exercise, we call attention to three inputs that, if substantially altered, can substantially change shortfall estimates. First, prevalence estimates (Table 2) may be greater than those used in this report, given that prevalences of MNS disorders are often under-reported. Second, a selection of target treatment service coverage for each MNS disorder depends on detectability and cultural-specific factors about willingness to seek care. Third, assumptions of worker productivity (that is, that a provider can treat 11 patients per day) may vary dramatically across country contexts. These inputs, as well as other aspects of the model-based estimates, should be carefully evaluated and refined by the KSA Ministry of Health. It is anticipated, however, that refinements to these inputs might yield an even greater shortfall of health care workers to treat MNS disorders than those reported here.

## Figures and Tables

**Fig. 1:**
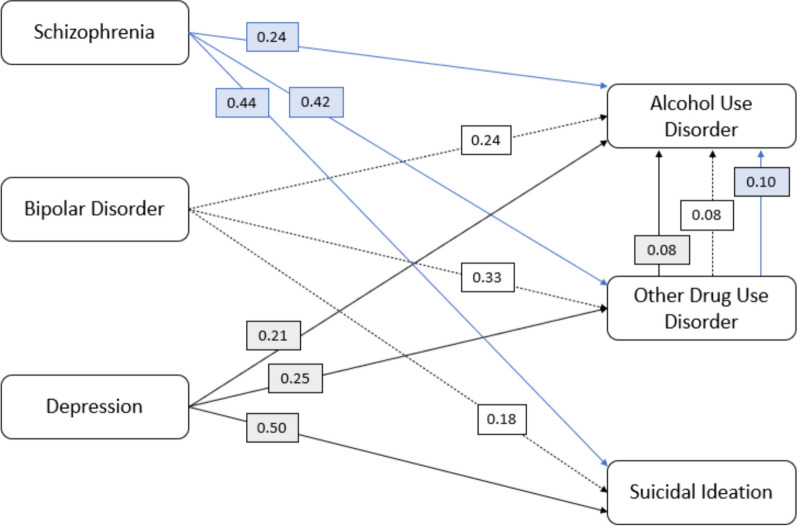
Overview of comorbidities: adjustments and direction of adjustment. Sources: Schizophrenia and alcohol use disorder [80]; other drug use disorders [80]; suicidal ideation [87]. Bipolar disorder and alcohol use disorder [79]; other drug use disorders [79]; suicidal ideation [88]. Depression and alcohol use disorder [81]; other drug use disorders [81]; suicidal ideation [88]. Positive predictive values for schizophrenia and alcohol use disorder (0.55); other drug use disorders (0.45); suicidal intention (0.79); [86, 89, 90]. Bipolar disorder and alcohol use disorder (0.35); other drug use disorders (0.35); suicidal intention (0.86): [90, 91]. Depression and alcohol use disorder (0.35); other drug use disorders (0.35); suicidal intention (0.86): [90, 91]

**Fig. 2:**
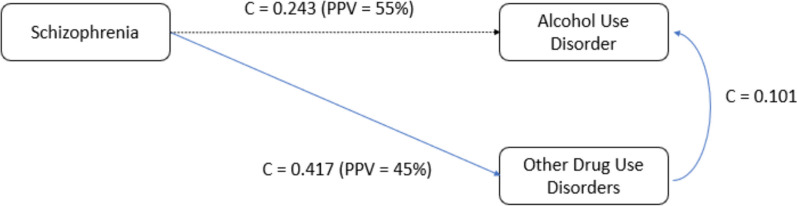
Example of comorbidity adjustment and positive predictive value for Schizophrenia, alcohol use disorder, and other drug use disorders. *Source:* Original figure for this publication

## Data Availability

All data generated or analyzed during this study are included in this published article [and its supplementary information files]. All data available in Tables 2, 3, 10, and C1. Detailed descriptions of the data sources are available in Table 1 and Appendices A1, B1, and C1.

## Appendix A

### Description of data sources

This appendix details the data sources, ranking, and prioritization used to arrive at the final prevalence estimates.

### WHO World Mental Health Surveys

The WHO’s WMH Surveys use a multistage cluster household probability sample with case–control design. Respondents were recruited from the General Authority for Statistics 2010 census for Saudi Arabia. Trained interviewers carried out a fully structured diagnostic interview using the WMH-CIDI. In Part 1, a core diagnostic assessment was carried out to quantify the prevalence of mental health conditions of primary interest. Part 2 assessed correlates and disorders that were of secondary interest. Respondents who met criteria for any disorder in Part 1, plus a subsample of 25 percent of respondent controls who did not meet any criteria, were included in Part 2. Post-assessment weighting was applied to adjust survey results for sociodemographic and geographic variables.

When comparing the results of the Saudi WMH Survey to other high-income countries, there are a few notable differences. The lifetime prevalence for mood disorders and drug abuse was higher at 6.8% and 1.4% vs. 5.2% and 0.5% for other high-income countries [73]. The higher prevalence of mood disorders can be attributed to bipolar disorder [74]. Alcohol use disorder was lower than for other high-income countries [75].

### WHO Global Health Observatory

The WHO Global Health Observatory Mortality Database uses a combination of health service data, population surveys, civil registration, and vital statistics to produce country estimates of disease prevalence [76]. We used the age-specific crude death rates (5- and 10-year groups) for suicide and applied these rates to the UN Department of Economic and Social Affairs (DESA) Population count estimate “weights” for Saudi Arabia [55] to arrive at the count of age-specific deaths due to suicide for four age groupings (18–34, 35–49, 50–64, and 65 + years).

To assess the prevalence of suicide ideation and/or attempts, we multiplied the age-standardized rates of suicide by 20 [77]. This multiplier coheres with the literature in which most persons with suicidal ideation and or those attempting suicides do not complete.

This dataset is not without its limitations. The lack of registration is exacerbated by the cultural and legal concerns that make suicide as a cause of death a particularly sensitive issue which can result in under-reporting and/or misclassification of deaths [76, 78].

### World Alzheimer’s report

The burden of dementia is expected to increase as the population ages and mortality due to communicable diseases decreases. Current dementia estimates for developing countries suggest that the prevalence of dementia is lower than in developed countries [45]. We used the World Alzheimer Group’s regional Middle East and North Africa estimates. The estimates of prevalence were available in six age groups five 5-year age groups from age 60 to 84, plus 85 and older.

Since the risk of dementia increases with age, we used UN population estimates [55] to calculate a standardized rate of dementia for individuals aged 65 and older. High-quality dementia prevalence studies were available in the World Alzheimer’s report, primarily in Western countries. A meta-analysis was conducted to estimate regional prevalence, and in regions where high-quality studies were rare, expert consensus (from the Delphi Consensus and Dementia Working Group) was also included. Few studies estimating dementia have been conducted in low- and middle-income countries [45]. For the Middle East and North Africa, only two empirical studies were considered.

### Global burden of disease

When estimates of prevalence using the above resources were not available or were not in line with prior workforce estimates, we used estimates from the GBD study. We used the Global Burden of Disease estimates for schizophrenia, child intellectual development disorders, childhood conduct and behavioral disorders, and childhood emotional disorders [54].

## Appendix B

See Tables 10, 11, 12

### Target coverage and estimates for service coverage, utilization, and staffing

As an update to Chisholm et al. [57], we consulted Dan Chisholm, an expert in health economics for the WHO, for an updated set of inputs for cost and impact of scaling up for mental health. In recent publications, treatment models were separated by basic, moderate, and intensive treatment of depression based on severity of the condition [58]. After reviewing the two publications from 2007 and 2016, we noted that there were substantial changes to the categories of resource usage (for example, inpatient, outpatient, residential care, and day care); estimates for service use inputs (for example, bed-days and visits/sessions), and estimated service coverage. At a minimum, the new inputs would yield workforce estimates that are substantially higher than prior estimates [35], which would not allow for a comparison of current and historical workforce projections. Therefore, we used the estimates, resource utilization, and service coverage from Chisholm et al. [57].

**Table 10:** Target coverage for target populations for priority conditions

Condition	Target coverage percent
Schizophrenia^a^	80
Depression^a^	33
Suicide^b^	80
Epilepsy^c^	80
Dementia^d^	80
Alcohol use disorder^a^	25
Other drug use disorders^e^	50
Childhood disabilities^f^	20

Sources*:*^a^[57]

^b^[78]

^c^[59]

^d^[60]

^e^[35]

^f^Taken from level attainable in developed countries [56, 61]

^g^Chisholm et al. [58] using treatment coverage for anxiety disorders

**Table 11:** Target estimates for service coverage and resource utilization

Service	Schizophrenia			Bipolar disorder			Major depression			Hazardous alcohol use		
	Service coverage %^a^	Average rate of use^b^	Resource use per case^c^	Service coverage^a^	Average rate of use^b^	Resource use per case^c^	Service coverage %^a^	Average rate of use^b^	Resource use per case^c^	Service coverage%^a^	Average rate of use^b^	Resource use per case^c^
Inpatient and residential care^d^												
Mental hospital care (long stay)	2	90	1.8	1	90	0.9	0	0	0	0	0	0
Community residential care (long stay)	2.5	180	5	1.5	180	2.7	0.5	90	0.5	0	0	0
Community psychiatric unit (acute care)	15	28	4.2	10	28	2.8	2	14	0.3	2	5	0.1
Outpatient and day care^e^												
Day care services	7.5	100	7.5	3	100	3	1	50	0.5	0	0	0
Hospital outpatient service	50	12	6	40	12	4.8	20	7	1.4	10	2	0.2
Primary health care: treatment	30	6	1.8	30	6	1.8	30	7	2.1	0	0	0
Primary health care: screening^f^	0	0	0	0	0	0	7–14	1	n.a	2–4	1	n.a
Psychosocial treatment^g^	30	8	2.4	30	8	2.4	20	6	1	25	3	0.8

Source [57]

n.a.: not applicable

^a^*Service coverage* = percentage of patients in the population expected to use the service or resource over course of 1 year

^b^*Average rate of use* = mean rate of uptake per year among those expected to use the service or resource

^c^*Resource use per “average” patient in the population* = percentage of patients expected to use the resource (coverage) multiplied by the average rate of use

^d^Average rate of use in days

^e^Average rate of use in visits

^f^Refers to coverage in the total adult population (before diagnosis)

^g^Index therapies used: family therapy (schizophrenia); problem-solving treatment (bipolar disorder); brief psychotherapy (depression); brief physician advice (alcohol use). Sessions last 40 min each, except brief interventions for alcohol use, which take 10 min

**Table 12:** Staffing proportions by health care setting and country income classification

Occupation	Outpatient		Inpatient	
	Day care (%)	Acute and primary care (%)	Acute care (%)	Long stay/residential care(%)
Low-income countries				
Psychiatrists/specialists	0.00	1.67	6.25	7.69
Nursing care provider	66.67	20.83	62.50	61.54
Psychosocial care provider	33.33	77.50	31.25	30.77
TOTAL	100.00	100.00	100.00	100.00
Middle-income countries				
Psychiatrists/specialists	0.00	3.57	10.00	6.67
Nursing care provider	62.50	28.57	60.00	66.67
Psychosocial care provider	37.50	67.86	30.00	26.67
Total	100.00	100.00	100.00	100.00

Source [57]

## Appendix C

See Figs. 1, 2

### Comorbidity adjustments

Bipolar disorder, depression, and schizophrenia have a high likelihood of comorbidity with alcohol and other drug use disorders [79–81] and suicidality [82]. Co-occurrence of these mental, neurological, and substance use conditions does not necessarily mean that each condition requires a separate dedicated treatment model. Some therapies and treatment models, administered within single visits with health workers, can effectively address two co-occurring conditions [83–85]. In the case of depression comorbid with alcohol or substance use, cognitive behavioral therapy and some medications can, within the same treatment model, address both conditions [83, 84]. The same holds for schizophrenia that co-occurs with substance use disorder [85].

Figure 1 illustrates the conditions and the hypothesized temporal sequence of onset of comorbidities. This overview illustrates the possible co-occurrence of conditions. The numeric values represent the proportion of persons who have the comorbidity associated with the respective path that connects two conditions. To walk through an example, 41.7 percent of individuals with schizophrenia have comorbid substance use [80]. This comorbidity is captured in Fig. 1 via the arrow connecting schizophrenia and other drug use disorders. The value 0.42 (rounded) means that 41.7 percent of individuals with schizophrenia have comorbid substance use.

When two conditions were comorbid with each other, we prioritized the condition with the higher target coverage. In the case of schizophrenia and other drug use disorders, we considered only other drug use disorders within schizophrenia (target coverage 80 percent) and not schizophrenia within other drug use disorders (target coverage 50 percent). After adjusting for comorbidities, we added a screening component to account for potential difficulties in screening for and detecting comorbid conditions within the target population. The primary measure considered was the positive predictive value. The positive predictive value is the likelihood that a standardized screening instrument is able to detect a comorbid condition within the target population.

To continue with the prior example, the detection of substance use in individuals with schizophrenia has a positive predictive value of 45 percent [86]. This means that, if the individuals with schizophrenia in our target population were all screened for substance use, there is a 45 percent chance that those who actually have comorbid substance use would be correctly identified or successfully screened. So, a 0.41 percent age-standardized prevalence of schizophrenia with 80 percent target coverage corresponds to 114,189 target cases within the total population. Within this group, we are assuming that 41.7 percent have comorbid substance use [80] and that 45 percent of this group can be identified for treatment [86]. This leaves us with 21,428 individuals with schizophrenia with detectable other drug use disorders, which means that we can remove 21,428 patients from our “other drug use” treatment model and potentially treat them within the schizophrenia treatment model.

To address comorbidity of schizophrenia, bipolar disorder, and depression with alcohol and other drug use disorders, we added another level of adjustment for these conditions. For each condition, we identified comorbid other drug and alcohol use [79–81]. In Fig. 1, the three arrows leading from other drug use disorders to alcohol use disorder depict these comorbidities according to condition. Using the same methodology as before, we assigned the comorbidity to the condition with the larger treatment coverage, which in this case was other drug use disorders (target coverage 50 percent, compared with 25 percent for alcohol use disorder). This means that alcohol use disorder can be treated under the other drug use treatment model. This effectively removes the double count of individuals with schizophrenia who have alcohol use disorder and other drug use disorders and allows us to independently assess alcohol use within schizophrenia.

Figure 2 walks through the example using schizophrenia and comorbid other drug and alcohol use disorder.

*Step 1*—Review treatment models for comorbidity of other drug use within schizophrenia that can be treated as part of schizophrenia treatment.

- 41.7 percent of individuals with schizophrenia have comorbid other drug use, and if screened, 45 percent of them can be successfully identified for follow-up.

*Step 2*—Quantify comorbidity of alcohol use disorder that can be treated within other drug use disorders. Remove from alcohol use disorder.

- 10.1 percent of individuals with schizophrenia with comorbid other drug use are likely to have an alcohol use disorder.

*Step 3*—Remove comorbidity of alcohol use disorder from other drug use disorders (within individuals with schizophrenia).

- This 10.1 percent is removed from the individuals with schizophrenia with comorbid alcohol use population and assigned to individuals with schizophrenia with comorbid other drug use to avoid double count.

## Appendix D

### Sensitivity analyses

*Scenario 0*: Base scenario using GBD prevalence estimates for all Childhood conditions.

**Table:**

Age group	Childhood intellectual disabilities (%)	Childhood conduct/behavioral disorders (%)	Childhood emotional disorders (%)
0–14	1.36	2.75	0.69

The base scenario requires a total of 17,128 mental health workers (49.2 per 100,000) or 1047 psychiatrists, 9440 nurses, and 6641 psychosocial providers.

*Scenario 1*: Uses the prevalence estimate for Childhood Intellectual Disabilities from Eapen et al.’s [43] study and GBD estimates for Childhood Conduct/Behavioral Disorders and Childhood Emotional Disorders.

**Table:**

Age group	Childhood intellectual disabilities (%)	Childhood conduct/behavioral disorders (%)	Childhood emotional disorders (%)
0–14	0.29	2.75	0.69

When the childhood intellectual disabilities prevalence is reduced by 79% from 1.36 to 0.29%, the total estimated workforce requires a total of 17,103 mental health workers (49.1 per 100,000) or 1046 psychiatrists, 9435 nurses, and 6622 psychosocial care providers.

A 79% reduction in the prevalence of childhood intellectual disabilities would mean that 25 fewer healthcare workers (0.1 per 100,000 or 1 psychiatrist, 5 nurses, and 19 psychosocial care providers) are needed to treat the new prevalence of disease.

*Scenario 2*: Uses the Childhood Conduct/Behavioral Disorders prevalence estimate from Mohammed et al.’s [92] study and GBD estimates for Childhood Intellectual Disabilities and Childhood Emotional Disorders.

**Table:**

Age group	Childhood intellectual disabilities (%)	Childhood conduct/behavioral disorders (%)	Childhood emotional disorders (%)
0–14	1.36	9.58	0.69

When the childhood conduct/behavioral disorders prevalence is increased by 248% from 2.75 to 9.58%, the total estimated workforce requires a total of 17,481 mental health workers (50.2 per 100,000) or 1054 psychiatrists, 9523 nurses, and 6904 psychosocial care providers.

A 248% increase in the prevalence of childhood conduct/behavioral disorders would mean that 353 more healthcare workers (1.1 per 100,000 or 7 psychiatrists, 83 nurses, and 263 psychosocial care providers) are needed to treat the new prevalence of disease.

*Scenario 3*: Uses the Childhood Emotional Disorders prevalence estimate from Mohammed et al.’s [92] study and GBD estimates for Childhood Intellectual Disabilities and Childhood Conduct/Behavioral Disorders.

**Table:**

Age group	Childhood intellectual disabilities (%)	Childhood conduct/behavioral disorders (%)	Childhood emotional disorders (%)
0–14	1.36	2.75	7.68

When the childhood emotional disorders prevalence is increased by 1013% from 0.69 to 7.68%, the total estimated workforce requires a total of 17,592 mental health workers (50.5 per 100,000) or 1067 psychiatrists, 9633 nurses, and 6892 psychosocial care providers.

A 1013% increase in the prevalence of childhood emotional disorders would mean that 464 more healthcare workers (1.4 per 100,000 or 20 psychiatrists, 193 nurses, and 251 psychosocial care providers) are needed to treat the new prevalence of disease.

*Scenario 4*: Uses the Childhood Intellectual Disabilities prevalence estimate from Eapen et al.’s [43] study and Childhood Emotional Disorders and Childhood Conduct/Behavioral Disorders prevalence estimate from Mohammed et al.’s [92] study.

**Table:**

Age group	Childhood intellectual disabilities (%)	Childhood conduct/behavioral disorders (%)	Childhood emotional disorders (%)
0–14	0.29	9.58	7.68

When childhood conditions are changed, total estimated workforce requires a total of 17,920 mental health workers (51.5 per 100,000) or 1,073 psychiatrists, 9710 nurses, and 7137 psychosocial care providers.

A 79% reduction in childhood intellectual disabilities, 248% increase in childhood conduct / behavioral disorders, and a 1013% increase in the prevalence of childhood emotional disorders would mean that 792 more healthcare workers (2.3 per 100,000 or 26 psychiatrists, 270 nurses, and 496 psychosocial care providers) are needed to treat the new prevalence of disease.

## Abbreviations

DALY: Disability-adjusted life year
DSM-IV: Diagnostic and Statistical Manual of Mental Disorders
FTE: Full-time equivalent
GBD: Global Burden of Disease
GP: General practitioner
ICD-10: International Classification of Diseases
KSA: Kingdom of Saudi Arabia
mhGAP: Mental Health Gap Action Programme
MENA: Middle East and North Africa
MNS: Mental, neurological, and substance abuse
WMH: World Mental Health
WMH-CDI: World Mental Health Composite International Diagnostic Interview
WHO: World Health Organization

## Footnotes

1 One DALY can be thought of as one year of life lost due to disability or premature death. This measure quantifies the health gap between the ideal health state—a theoretical state in which mortality is caused only by old age—and years of life spent disabled/injured in subpar health due to disease. DALYs are often used as a basis for health policy making as well as for setting intervention priorities.

2 In absence of a probability sample of households or individuals, a community-based study that selected individuals for participation through a community register was preferred. Case control was necessary for inclusion.

3 For childhood intellectual disabilities, the Eapen et al. study found a prevalence of 0.29 percent for intellectual disabilities. This is 4.7 times lower than GBD estimates at 1.36 percent [43]. For childhood emotional disorders, the Mohammadi et al. [44] study found a prevalence of 9.58 percent for childhood conduct/behavioral disorders and 7.86 percent for childhood emotional disorders. These estimates were 3.4 and 13.9 times higher than GBD estimates (2.75 percent and 0.69 percent, respectively).

4 The prevalence (1.11 percent) shown is rounded down from (1.11256576431905 percent).

5 The unrounded FTE are 71.28 FTE which includes 1.19 psychiatrists, 14.85 nurses, and 55.24 psychosocial care providers.

6 This calculation 1,153,051 is rounded to the nearest 100. For virtually all remaining figures that exceed 1000, we rounded to the nearest 100.

7 This calculation at 1,957,393 was rounded to the nearest 100 and was derived using the population prevalence for bipolar disorder at 1.07 percent and target coverage at 80 percent.
